# Sensations, symptoms, and then what? Early bodily experiences prior to diagnosis of lung cancer

**DOI:** 10.1371/journal.pone.0249114

**Published:** 2021-03-29

**Authors:** Britt-Marie Bernhardson, Carol Tishelman, Birgit H. Rasmussen, Senada Hajdarevic, Marlene Malmström, Trine Laura Overgaard Hasle, Louise Locock, Lars E. Eriksson

**Affiliations:** 1 Division of Innovative Care Research, Department of Learning, Informatics, Management and Ethics, Karolinska Institutet, Stockholm, Sweden; 2 Stockholm Health Care Services (SLSO), Stockholms County Council (SLL), Stockholm, Sweden; 3 Department of Health Sciences, Lund University, Lund, Sweden; 4 The Institute for Palliative Care, Lund University and Region, Skåne, Sweden; 5 Department of Nursing, Umeå University, Umeå, Sweden; 6 Department of Public Health, Research Centre for Cancer, Diagnosis in Primary Care, Research Unit for General Practice, Aarhus University, Aarhus, Denmark; 7 Health Services Research Unit, University of Aberdeen, Aberdeen, United Kingdom; 8 Nuffield Department of Primary Care Health Sciences, Health Experiences Research Group, University of Oxford, Oxford, United Kingdom; 9 School of Health Sciences, University of London, London, United Kingdom; 10 Department of Infectious Diseases, Karolinska University Hospital, Huddinge, Sweden; Chinese Academy of Medical Sciences and Peking Union Medical College, CHINA

## Abstract

Lung cancer (LC) generally lacks unique core symptoms or signs. However, there are a multitude of bodily sensations that are often non-specific, not easily understood, and many times initially not recognized as indicative of LC by the affected person, which often leads to late diagnosis. In this international qualitative study, we inductively analyzed retrospective accounts of 61 people diagnosed with LC in Denmark, England and Sweden. Using the bodily sensations they most commonly spoke about (tiredness, breathlessness, pain, and cough), we constructed four sensation-based cases to understand the pre-diagnostic processes of reasoning and practice triggered by these key indicators of LC. We thereafter critically applied Hay’s model of sensations to symptoms transformation, examining its central concepts of duration, disability and vulnerability, to support understanding of these processes. We found that while duration and disability are clearly relevant, vulnerability is more implicitly expressed in relation to perceived threat. Tiredness, even when of long duration and causing disability, was often related to normal aging, rather than a health threat. Regardless of duration, breathlessness was disturbing and threatening enough to lead to care-seeking. Pain varied by location, duration and degree of disability, and thus also varied in degree of threat perceived. Preconceived, but unmet expectations of what LC-related cough and pain would entail could cause delays by misleading participants; if cough lasted long enough, it could trigger health care contact. Duration, disability, and sense of threat, rather than vulnerability, were found to be relevant concepts for understanding the trajectory to diagnosis for LC among these participants. The process by which an individual, their family and health care providers legitimize sensations, allowing them to be seen as potential symptoms of disease, is also an essential, but varying part of the diagnostic processes described here.

## Introduction and aim

Lung cancer (LC), with its’ still increasing incidence, is the most common cancer diagnosis globally with approximately 2 million new cases per year [1]. Although survival rates may have improved, recent comparable statistics are hard to find; in 2011 the overall five-year survival was as low as 16.3% in Sweden, 10.9% in Denmark, and 8.8% in England [2]. LC has been referred to as a silent disease [3] as it lacks unique core symptoms or signs that may function as early indicators, such as a lump signalling breast cancer; haemoptysis may be considered an exception, as it is strongly correlated with LC, although is relatively rare as a first symptom [4]. However, there are a multitude of bodily sensations that are often non-specific, not easily understood, and many times initially not recognized as indicative of LC by the affected person or healthcare professionals. Recognition of these factors may influence patients’ chances of curative treatment and survival (e.g. [4–7]).

A complicating factor in interpreting sensations is that smokers are less likely to seek help for respiratory symptoms than non-smokers [8–10]. Even when LC is suspected, delay in seeking help has been shown to be related to stigma, self-blame, and the belief that others blame the smoker for having caused the disease themselves [11–13].

The lack of clear symptomatology specific to and indicative of LC, and the unclear origin of experienced sensations and symptoms such as tiredness, breathlessness, pain, and cough, are confirmed by existing research on people’s reports of symptoms observed prior to LC diagnosis [5, 7, 14–21]. This research has generally focused on which symptoms occur before diagnosis, catalyze care-seeking behavior, and/or may be predictive of LC. Although empirical insight into how people reason about, process, interpret, and organize different bodily sensations into potentially relevant symptoms prior to their LC diagnosis is sparse, insight into these experiences may contribute to endeavors to facilitate earlier diagnosis. In the present study, we therefore focus on the phase in which people later diagnosed with LC initially perceive bodily sensations. We explore these individuals’ retrospective accounts of how these perceived sensations related to four key indicators of LC—tiredness, breathlessness, pain, and cough, focusing on the processes of reasoning and practice these sensations triggered prior to LC diagnosis. We also explore the extent to which Hay’s model of sensations to symptoms transformation [22] might serve to support understanding of these processes.

## Background

There is a growing body of literature regarding bodily changes. One common feature in this literature is that interpretation of bodily sensations depends on socio-cultural context [23]. A bodily sensation is defined as an embodied, lived, and felt experience [22, 24], in contrast to a symptom, which results from a constructed and socially informed interpretive process, often trigged by an embodied sensation [22]. Interpreting a bodily sensation and recognizing it as a symptom is a process of negotiation shaped by many factors, e.g. previous experiences of sickness and contact with healthcare services [25–27]. Such inner negotiation is in part dependent on a person’s beliefs; Pedersen et al. [28] showed that “negative cancer beliefs”, e.g. perceiving cancer as a death sentence, were associated with an increased likelihood of a longer time interval before seeking help. Furthermore, the person’s ability to verbalize [29, 30] and discuss [25, 31] the sensation with others is central in the process of seeking social legitimization for a sensation [22]. In order for an individual to feel that their sensation is in line with a socio-culturally accepted description of a recognizable symptom, healthcare professionals also need to interpret the reported bodily sensation as meaningful [22, 32] thus allowing the person to become a ‘candidate’ for medical attention [22, 25, 29, 32].

Based on fieldwork in Indonesia, Hays [22] suggests a model by which sensations are interpreted, become pathologized, and are social legitimized as symptoms. According to Hay’s model, crucial concerns that trigger the transformation of sensations into symptoms include experiencing them as being of longer duration than expected; being disabling beyond what could be considered normal; and heightening one’s sense of vulnerability (p. 222). Despite the radical difference in context, and while recognizing the important role played by different cultures in the processes of transforming sensations to symptoms, we argue here that several aspects of Hay’s model are applicable and relevant in the western European contexts included in the present study.

## Methods and design

The data analyzed in the present study derive from an explorative international qualitative study investigating patients’ experiences of their trajectories to receiving a LC or colorectal cancer diagnosis and initiating treatment (N = 155), conducted in Denmark, England, and Sweden [33]. This article is based on data from the sub-sample of patients diagnosed with LC (n = 72). These countries, with the significant variation in LC survival rates noted above, participated in the International Cancer Benchmarking Partnership (ICBP) [34]; the explorative international qualitative study was originally intended as a complement to ICBP’s predominately quantitative patient data.

The present study was conducted in accordance with the varied ethical regulations in each country Regional Ethics Board, Lund, Sweden, reference 2014/819 Research Ethics Service reference 14/NS/1035, England. In Denmark, we also followed the existing country specific regulations and research norms. This entailed meeting the criteria established by the Danish Data Protection Agency (J.2016-41-4662) for non-invasive research with human subjects and following the standard ethical protocol of the American Anthropological Association [35]; the Danish Research Ethics Committee System Act only provides ethical clearance for studies using human biological materials.

### Recruitment

Adult patients with LC were recruited within six months of diagnosis in Denmark, England and Sweden. Purposeful sampling was used to achieve variation across gender, age, urban and rural residence, and trajectories to diagnosis. Recruitment was primarily via contact with hospitals responsible for treatment in each country; in England and Denmark this was complemented by recruitment via support groups, social media, and word-of-mouth. Written and oral information about the study was provided with the opportunity to ask questions before agreeing to participate, with all procedures in line with the norms and regulations in each participating country.

### Data collection and participants

A semi-structured interview guide was developed jointly by researchers in the three countries to maximise data comparability (for more detail about this process, see [33, 36]). In 2015, interviews with participants were held, generally in their homes unless another location was preferred, by researchers with backgrounds in anthropology (Denmark), sociology (England), and nursing (Sweden).

The conversational interviews began with the open-ended question: *Please begin by telling me*, *in your own words and in as much detail as you want*, *about everything that has happened since you first started to suspect there might be a problem with your health*? Examples of follow-up probing questions include "when were you first aware of this?" and "at what point did you think this might be serious?" While responses could include a wide variety of information, in the present analysis we focus on responses related to bodily signs and sensations (see [12, 33, 37–39] for other publications from this study). All interviews were audio-recorded and professionally transcribed verbatim in their original language. Preliminary analysis began in each country in conjunction with data collection. The research team held monthly telephone discussions about the data obtained and stopped recruitment when the team felt that an adequate number of participants generating robust data had been collected from each country [40].

### Analysis

Interviews were first analyzed in their original language using NVivo 10 by the researchers in each country who sorted the data into general areas related to the topic guide, in this case data related to bodily sensations, symptoms, and signs which were then merged into a combined dataset. This combined dataset was then analyzed inductively by bilingual researchers to search for commonalities, patterns and contradictions. The quotes selected for illustration were then translated into English when necessary.

The first author (XX) repeatedly read and began a preliminary inductive analysis of the combined dataset. Analysis was furthered through regular discussions in the Swedish research group, with feedback from our international colleagues. We noted that there were four bodily sensations that were most commonly spoken about by these participants as salient: tiredness, breathlessness, pain, and cough; this finding also resonated theoretically with the literature and clinical experience. We were inspired by Sandelowski’s description of “casing” [41] in our continued analysis. This approach allowed us as researchers to further define our focus by constructing four sensation-based cases based on the above sensations through an iteractive and theory-laden process, rather than by examining individuals as cases or generating more inclusive themes. We then turned in-depth analytic focus to each participant’s narratives around each sensation-based case, moving iteratively between data from individuals and the generated cases. This form of analysis using a case-oriented instead of a variable-oriented analysis provided a means to manage complexity in our data while maintaining empirical intimacy [41]. Hay’s model did not influence analysis but was rather applied after analysis was completed as a means to understand our results.

In the presentation of findings below, quotations from participants have been selected for illustration. False starts, repeated phrases, and irrelevant information have been omitted from quotes as indicated by /…/. All names are pseudonyms, with the participant’s country shown in parentheses.

## Results

These results are based on 22 interviews with patients in Denmark, 20 in England, and 30 in Sweden. Among these, 11 participants (4 Denmark, 2 England, and 5 Sweden) did not report experiencing any sensations leading to diagnosis; in these cases, diagnosis occurred fortuitously during contact with healthcare services for other reasons, e.g. a heart attack or a yearly checkup. The dataset underlying this analysis thus consists of data from 61 participants aged between 31 and 90 years, with 45% women.

The results are organized as bodily sensation-based cases, illustrating and contrasting the described trajectories for tiredness, breathlessness, pain, and cough and considering variation and patterns both within and among these cases. As noted above, the underlying sensations and symptoms were those described as occurring first or spoken of as particularly burdensome, notable, or important by these participants. While there were reports of other sensations and symptoms in these data, e.g. appetite loss, night sweats, and hoarseness, participants did not describe these as equally salient as those chosen for the cases.

As previously noted, LC symptomatology is complex. Prior to discussing the four cases, we highlight an overarching challenge and cause of uncertainty described by several participants, related to interpreting and acting on sensations which later proved to be related to LC. Alice (England), contrasted her initial experiences which led to a LC diagnosis, with those leading to her earlier breast cancer diagnosis:

*“See*, *like with my cancer*, *my both cancers*, *right*, *with the lung cancer*, *I haven’t*, *it never entered my head but with breast cancer*, *I knew it was breast cancer because I could feel the lump*. *And I went to [*husband*]*, *I said*, *‘This isn’t right*.*’ And I*, *I felt it*, *it was there*. *But with lung cancer I had this cough*, *I’ve had coughs before*, *you know*, *‘cause of chest infections*. *But I never thought it would be lung cancer*. *Never even*, *it never even entered my head*.*”*

Whereas Alice described an unusual lump which led her to suspect breast cancer, her cough was a more familiar experience, which might have a variety of causes. Alice was thus greatly surprised to learn that she had LC; she had not considered her cough as reason enough to seek medical assistance.

### The case of tiredness

Tiredness was unique in the length of time it remained a sensation before being recognized as a symptom of sickness. Most participants did not at first interpret their tiredness as sickness-related at all, and it rarely catalyzed contact with the health care system. Alex (Denmark) was one of few participants who did contact the healthcare system because he was tired.

*“Yes*. *On* [names date] *I went to the doctor*. *I did it because*, *I always work on something and I had a project going*, *and that spring I couldn’t—damn it—get started*. *Then* [wife] *says to me*, *‘Either you get started*, *or you go to a doctor’ and then I had to go to a doctor”*.

Harriet (England) was more typical of those interviewed here in that she did not consider consulting her physician for her tiredness, talking about how she first related it to her (negative) expectations of ‘normal’ aging:

“*I probably did feel more tired before I even had the cough, if you see what I mean. But you don’t even think, you don’t think of that as being a symptom, you just think that’s getting older, you know*.”

Harriet was not alone in this interpretation; Karin (Sweden), in her early 60s, described a long diagnostic process with several hospital stays due to pneumonia. She spoke of tiredness which impacted so greatly on her daily life that she decided to retire early, but still did not reflect on it as something potentially pathological:

“*And then I was discharged from the hospital. But I’d had time to think when I was there, so as soon as I came home, I said to my husband, ‘now it’s enough already, I don’t have the energy to work. I’m quitting. I must have suddenly gotten old’. Because I was so horribly tired, both during the daytime and evenings and all the time, you know. So I quit*.”

Harriet and Karin were not exceptional in referring to tiredness as a sensation indicative of ‘normal’ aging, rather than an abnormal disease process. Harriet retrospectively recalled an incident that might have led her to reconsider this:

*“I can remember thinking that*, *it’s a silly thing*, *but I was standing in the garden and that must have been sort of late spring*, *I think*. *And my next-door neighbor was out*, *you know*, *busily gardening and I just was feeling really weary and thinking*, *gosh*, [neighbor’s] *ten years older than me and she’s full of energy*, *you know*.*”*

Despite this comparison with her older neighbor, Harriet did not reevaluate her tiredness as a symptom worthy of further exploration. For her, as with others, the sensation of tiredness rarely was seen as a symptom prior to diagnosis and contact with the health care system was generally triggered by other things. Karin was diagnosed after a lung x-ray as part of a post-pneumonia checkup, and Harriet due to her persistent cough.

To summarize in relation to Hay’s frameworks of sensation to symptom, in these interview descriptions, tiredness was often of long duration and could lead to disability in the sense of exerting high levels of interference on activities of daily life. However, it seemed not directly linked to a sense of personal vulnerability nor perceived as a threat, but was instead interpreted as related to aspects of life other than health/disease.

### The case of breathlessness

In comparison to tiredness, breathlessness seemed to be a sensation that often both developed and caused people to seek medical care rapidly. Elsie (England) noticed that she was suddenly breathless while walking the dog *“… literally on one day*. *It wasn’t sort of like a lead-up where I started thinking*, *‘Oh*, *something’s not right*.*’/…/ I thought*, *‘This isn’t right*. *I don’t*, *I shouldn’t feel like this’*.*”*

George (England) told the interviewer that *“there was no way that it [*breathlessness*] could have been ignored”*; he just had to go to the primary healthcare center. Several participants described breathlessness in terms of rapidly losing physical capacity, e.g. Richard (Sweden) succinctly indicated this, saying: *“One sort of lost…kind of like with a car that is missing a cylinder”*. This sensation was beyond what Richard was able to rationalize as normal.

David (Sweden), who described several symptoms, instead spoke about his breathlessness as gradually increasing. It was the reason he contacted the health care system and was diagnosed with LC:

*“… my friend and I*, *we started to take super-fast walks to get into shape*, *and that worked ok*. *And then during the fall*, *it felt like you weren’t getting anywhere*, *you were stuck even though I kept with it*, *had to change my breathing also* [breathes rapidly to explain]/…/ *It was kind of like the loss of capacity made me breath faster*. *But I didn’t think so much about it /…/ And then during the fall*, *it kept getting worse*, *just this breathing part*, *so finally whenever I inhaled I would start a cough reflex if I breathed in too much /*…*/ And then I went to the health center*, *because I never go to those places /…/ But they reacted maybe more to my heart and all that*. *They were a little perplexed but they were quick and /…/ did a lung x-ray and sent me to check my heart…*.*”*

The disabling sensation of breathlessness felt beyond what both Richard and David considered normal. For a few participants like David, the onset of breathlessness developed gradually over time. In these cases, the sensation of breathlessness was reconceptualized as a symptom—in Richard’s case due to the disability caused, while for David, duration also played a role.

Whereas the breathlessness experienced by both Richard and David was legitimized by medical tests, Astrid (Sweden) had a different experience:

*“So then I thought there was some kind of resistance when I breathed*, *and I felt that there was something that wasn’t right*, *and I had to do these tests where I had to exhale and all that*. *But there was nothing special*, *they just said it was imagination*, *they said*, *or they didn’t really say that*, *but just that there was nothing wrong*. */…/ And then I had to be satisfied with that* [laughs] *because the test results were ok and all*.*”*

Astrid did not receive an explanation for her breathlessness, and thus, no legitimization for her experience at this point. The physician acted in line with the test results rather than her description of her bodily experience. It wasn’t until a yearly control x-ray found exacerbation of a previously known chronic lung change that the health care system initiated a diagnostic process for LC.

In a few outlying cases, breathlessness did not in itself lead to contact with the healthcare system. Mikael (Sweden) described his breathlessness in conjunction with smoking without attributing it to an underlying sickness: *“So that’s why one didn’t react specially to it”*.

In summary, in line with Hay’s frameworks of sensation to symptom, breathlessness was generally disturbing to informants’ daily lives and perceived as a threat that might be implicitly interpreted as linked to a sense of personal vulnerability. In situations in which it developed gradually or changed over time, the process of legitimization as a symptom was less clear cut, many times demanding confirmation by objective medical tests.

### The case of pain

Pain was one of the most commonly reported early bodily sensations prior to LC diagnosis mentioned by the participants in this study. It was similar to breathlessness in that it often catalyzed contact with the health care system. However, pain was not always related to the lungs, and was often not seen as indicating LC by either the individual or health care practitioners. Ole (Denmark), who experienced chest pain when smoking, explained: “*So when I started to get real pain in my chest*, *I thought that it was something with my heart*.” Anders (Denmark) on the other hand, said: *“It felt like I had overstrained my back”*.

Eva (Sweden), who described pain as the first sensation causing her concern, spoke of how this led her to contact the healthcare system:

*“It started with me having pain in my right shoulder*. *I could hardly…I just could move my arm forward a little bit and then*, *‘what’s happening*?*’ Yeah*, *well I talked to Physiotherapist X and Doctor Y*. *And they said that I most likely had gotten an inflammation…*. *And so*, *I got* [an over-the-counter NSAID anti-inflammatory agent*] /*…*/ And so it got a little better but then my shoulder blade started to ache back here* [pointing] *and there was this cutting pain*, *continually*, *you could say*. */…/ this with my shoulder blade felt so strange because …it wasn’t normal aching*, *it felt like a sore*, *and so like shooting knives*.*”*

Once participants were aware that the pain they experienced was related to LC, they often reflected on its’ localization, sometimes explicitly expressing their assumption that LC pain would have been more directly related to the affected organ.

Considering it in retrospect, Eva described her pain as “*not normal*” and as a catalyst for her contacting the healthcare system. However, pain was often said to also be interpreted by professionals as of muscular origin or as a symptom of indigestion. Agnes (England) speaks of her difficulties in trying to convince healthcare providers that what she experienced was of a different quality than her previous muscle pain, saying: *“This pain is worse*. *I’m telling you*, *it’s not muscle damage*.*’ They said*, *‘It has to be muscle damage*, *your x-ray has come back clear*.*’”* The lack of agreement between the x-ray results and her experience proved difficult, making the very subjective nature of experiential knowledge of one’s own body clear. Eva, on the other hand, spoke about how she prepared herself to convince her care providers that her ‘non-normal’ shoulder pain should be further evaluated: *“*… *and I decided that this should damn well be checked out*. *So*, *I had thought that I would ask for a MRI [*Magnetic Resonance Imaging*] of my shoulder”*. However, she did not need to convince her health care providers, who referred her for an MRI which led to the LC diagnosis.

In summary, pain, as with tiredness, was often not interpreted as indicative of LC. While its’ characteristics could vary, it often led, as did breathlessness but not tiredness, to care seeking. Participants explained that they had expected there to be a more direct relationship between the location of the pain and the source of the problem. In relation to Hay’s frameworks of sensation to symptom, such referred pain could vary in relation to duration, degree of disability and threat, as well as link to a sense of personal vulnerability. Interpretation of this type of referred change appeared to challenge both the participants and their healthcare providers. Again, confirmation via medical tests was an important facilitator in a legitimization process.

### The case of cough

Cough differed in several ways from the other sensation-based cases. While it also was one of the most predominant bodily experiences in this group of informants, it is unclear if it can be considered a sensation, as it is not only embodied, lived and felt, but is also apparent to others. This may be expected to allow it to be perceived more readily as a symptom, but cough was still subject to constructed social processes through which its significance was interpreted.

It was common that participants described having a cough for an extended time period, something that tended to normalize its existence. Oscar (Sweden) exemplifies this, saying:

*“There was probably a bit of a cough*. *I’ve heard that/…/ ‘yeah*, *you did complain about your cough’*. *Did I*?*/…/I haven’t thought about it*. *It didn’t bother me so terribly*. *One may have …* [coughs], *just coughed like that*. *Not in that way that you nearly throw up*, *but just a cough*.*”*

Rachel (England) was typical of several informants as she described a cough that began in conjunction with a cold, and which she initially paid no mind to because “*everybody has it”*.

*“It was just*, *just about Christmas time*, *everybody had that dreadful lurgy flu or virus or whatever you want call it*. *And*, *of course*, *I had got a cough*, */…/ I’d think*, *gosh I’ve never had a cough last this long*. *So of course*, *hearing everybody else saying that*, *I thought*, *well*, *you know*, *I’ve got used to it*. *But it just went on and on* [coughs].*”*

In the case of both Oscar and Rachel, their interpretations of their cough as not signifying a threat were supported by those surrounding them; as Oscar notes, it was only retrospectively that those around him pointed out that he had complained about his cough.

Shortly before data collection, a public health campaign in England had encouraged people to seek care if they coughed for more than three weeks. Some of the English participants mentioned the campaign, but even so did not link their cough with the possibility of LC. As Laura (England) said:

“*And so, yeah I didn’t really consider lung cancer /…/I mean, I did remember hearing things ‘Go to your doctor if you’ve had a prolonged cough.’ But I imagined that a lung cancer cough would be coughing up blood and losing weight, feeling ill. And mine really was, it was nothing*.”

The notion that a LC cough would have particular characteristics could deter care-seeking behaviors. Other participants had other explanations for the long duration of their cough, which meant they did not see it as necessary to seek care. Carina (Sweden) said she initially thought the cough was from inhaling irritants when renovating her basement. Fiona (England) began to wonder if *“I might have an allergy to the dog”*.

For many their cough was nothing that seemed unusual or demanded special attention, however other participants could interpret the characteristics of their cough as more unusual, as Alice (England) said: *“it was a*, *a strange cough*. *It wasn’t a chest cough*. *As you’d think it would be*. *But it was all throat”*. However, despite this, Alice’s cough did not meet her expectations of the threatening nature of a cough she thought would be indicative of LC.

It is noteworthy that several participants mentioned *not* coughing up blood, implying that they believed that this would have been a clearer signal of LC. Hemoptysis was described by two of the 61 participants and was not an initial bodily sensation for either but in both cases occurred post-diagnosis.

Other participants who described a sudden onset cough. Anna (England) told us that she was perfectly healthy, playing golf three to four times a week before feeling *“a prickly throat”* in July. She described her shift in interpretation from *“it might be something”* to being *“absolutely something”* as occurring very quickly. She said she had a cough that drove both her and her husband *“up the wall”* and was thus irritating to the point of interfering with daily life. Anna said she considered *“Am I being a wimp*?*”*, explaining that she interpreted the cough as indicative of an infection rather than a symptom of LC: *“there were no symptoms* [of LC] *and it all came so out of the blue”*. In any case, her cough, though of relatively short duration, was disabling enough to stimulate her to contact the healthcare system, leading to diagnosis.

Rosa (England) was one of the participants with a long-duration cough that triggered contact with the healthcare system. She related her cough to smoking, saying:

*“I’ve not smoked for about seven or eight years—was a heavy smoker—on eighteen-twenty a day easy*. */…/ [*Now*] I had this persistent cough which I recognised as a smoker’s cough*, *like [*demonstrates cough*] and it was constant*. *I thought*, *‘I don’t bloody smoke*,*’ and now nobody smokes in the house /…/*. *So*, *but I had this cough and I thought*, *‘Right*, *go to the doctors*.*’ So*, *I went*.*”*

However, Rose also spoke later in the interview about how it was a combination of sensations that stimulated her to begin a diagnostic process, with not only her cough but also tiredness and breathlessness, making her feel “*like an old woman*”. Rather than normalizing this, she went to the local healthcare center.

As in the case of breathlessness and pain, when participants decided that their cough called for investigation by the healthcare system, the reaction of healthcare staff became crucial in the process of interpretation. Cough could be a symptom of a wide variety of conditions; in the case of Alice (England), her cough was initially interpreted by healthcare providers as an indication of asthma, before an x-ray showed LC. The physician who Bengt (Sweden) saw, related his cough to a lung infection although Bengt describes not being convinced by this explanation:

*“He listened to my heart and said that ‘you have a cold’*. *‘No’ I said*, *‘I don’t have a cold*. *I have a different kind of cough*. *It’s not a cold’*. *‘Yes*, *you have a cold’ he said*. *So*, *he prescribed xx* [names cough medicine] *and that was on Thursday*. *On the weekend*, *my wife started to talk about*, *‘what’s going on with you*? *You’re panting when you breathe’*. *And that same night I could*, *I had a hard time getting air when I breathed*, *so on Monday I went back there again*. *Booked*, *got an emergency time then and said ‘I don’t have a cold’ I said*. *‘This is something else’*.*”*

In Bengt’s case, when breathlessness compounded his cough, the sense of threat increased and he was persistent in again contacting the healthcare system, this time seeing it as urgent.

To summarize in relation to Hay’s frameworks of sensation to symptom, cough was often of long duration, which in and of itself was not enough to trigger contact with the healthcare system even when impacting on daily life, as a LC cough was expected to have special characteristics. When experienced in conjunction with breathlessness, or in the case of hemoptysis, the cough was interpreted as more threatening, and tended to trigger contact with the healthcare system. However, both the involved individual as well as healthcare professionals could initially have a range of explanations for a cough, prior to considering LC.

## Discussion

This international qualitative study complements the literature to date by providing a unique in-depth exploration of early experiences related to the interpretation of and responses to four key bodily experiences—tiredness, breathlessness, pain and cough—which may be indicative of LC. We used a sensation-based case approach to study these in the context of a LC diagnostic process [41]. We also considered the extent to which Hay’s [22] framework for sensations to symptoms transformation, in which duration, disability and vulnerability are central concepts, may support understanding of these interpretive processes. While duration and disability are clearly relevant in these data, we find no direct mention of personal vulnerability; however, this appears to be implicitly expressed instead in relation to the degree of threat perceived.

Based on this approach, we found that while tiredness could be of long duration and cause notable disability in the sense of interfering with daily life, it was rarely perceived as related to the domain of health and illness, and thus not as a threat, often related to a ‘normal’ aging process instead. Breathlessness of varying duration, generally disturbed life and was more likely to be perceived as a threat to health, and lead to health-seeking behaviors. Pain could vary by location, duration, and degree of disability, factors which each appear linked to the degree to which it was perceived as threatening. Cough differed from the other bodily experiences in that it was perceptible to others, and thus, according to Hay’s definition [22] might be considered a symptom, rather than sensation, from the onset. Cough and pain were similar many times in not meeting preconceived expectations of what they would entail if indicative of LC; referred pain was often not associated with lung ailments, and there were expectations that a LC-related cough would produce blood, which was rarely the case in these data. Despite long duration, cough was rarely seen as threatening when not in conjunction with other symptoms, although, if lasting long enough, it could trigger health care contact.

Tisheman et al [42] differentiated between symptom intensity and distress in people diagnosed with LC, and concluded that difficulties with breathing, and pain experiences, functioned as icons representing LC-associated threats. These symptoms were strongly associated with distress, even with low occurrence. Breathing appeared to symbolize life itself, making breathlessness particularly threatening. Pain appeared to be strongly feared and associated with cancer and cancer death. However, fatigue, the symptom reported as most intense, was found to be relatively non-threatening. These interpretations appear applicable even in the study presented here.

There are several other aspects of Hay’s [22] framework which are of relevance here, e.g. processes of social legitimization for sensations worrisome enough to be tentatively conceptualized as symptoms. These processes of negotiation occur at different levels—internally, among family, friends and in social relationships, and in contact with the health care system. One issue of potential concern here is what McLachlan et al [21] discuss in terms of alternative explanations, and which we see numerous examples of in our data. Alternative explanations seemed not the purview of the participants alone (see also Botey et al [43]), but according to these participants’ descriptions, healthcare providers could also suggest numerous other explanations and solutions, prior to considering LC. Alternative explanations by participants in this study are similar to those described by McLachlan et al [21], related to comorbidities and other benign phenomena, and most notably in the case of tiredness, related to expectations of normal aging. This difficulty in understanding tiredness is in line with Finsterer & Mahjoub’s review of fatigue [44], noting that people experiencing it are challenged in determining if the sensation is normal or abnormal, since it can be a common part of daily life, as Hannaford et al [45] also recently pointed out based on data from the UK. Hall et al [46] also found that, among patients with colorectal cancer, fatigue was particularly difficult to understand and often only recognized in hindsight.

Early work by Tishelman [47, 48] found that, among people already diagnosed with cancer in Sweden, ageing was often mentioned as an explanation for symptom experiences, particularly those that, as with tiredness, were diffuse or without clear readily available treatment. This was interpreted as a means of making sense of something un-understandable and non-normal, transforming experiences into more manageable, ‘normal’, and thereby less threatening, even if undesirable, developments. In the present study, aging was only used to explain tiredness, although Rose spoke of feeling like ‘an old woman’ when experiencing several symptoms at the same time. This differs from other research, indicating ageing as an explanation for other symptoms as well; Crane et al. [11] notes this in relation to breathlessness, and Murray [26] in relation to cough.

Other participants described other forms for normalizing sensations, e.g. a cough that was thought to originate from smoking or which they saw as chronic was incorporated into ‘normal’ everyday life. However, it is important to also recognize that our data is limited in that it is retrospective and thereby colored by later experiences, particularly the participants’ knowledge of having cancer when interviewed. Participants often described their sensations as minor and unremarkable in nature, implying a need to legitimize what might retrospectively be considered delayed care seeking, a phenomenon also reported by MacLean et al. [49]. Participants’ descriptions are also unable to be linked to objective disease characteristics, e.g. staging at diagnosis, with interviews instead indicating how participants try to understand, make sense of, and legitimize their previous actions or lack thereof, rather than considering the severity or extent of LC at diagnosis in relation to the participants’ stories. In addition, when considering the implications of these data, it should be remembered that a broader range of sensations and symptoms were described by the participants; by focusing on these four cases in-depth in an effort to further understanding of the most prominent sensations, other more discrete sensations are not addressed.

The fact that individuals are often reluctant to seek healthcare or talk about what they consider minor sensations and symptoms [11, 46] suggests a conundrum for the healthcare system. One important feature of Hay’s [22] model of sensation to symptom transformation, is that it circumnavigates issues of patient or provider competency in assessing an existing clinical reality, which then is or is not legitimized. Hay [22] instead focuses on processes by which experiences are transformed. Challenges in processes of interpretation that our participants describe undergoing in determining whether or not they should contact the healthcare system, seem to have a parallel in the interpretative processes that practitioners carry out in determining follow-up and potential relevance even for other patient groups. Previous research has shown that the ability to verbalize a sensation is crucial for its interpretation [29, 30], but we note in our data that participants may have difficulties in communicating the nature of their sensations. However complex it may be for practitioners to then determine reasonable follow-up plans, we see here, as Evans [50] pointed out, that timely actions by healthcare professionals appear central to avoiding frustration and a sense of not being taken seriously (see also e.g. [51–53]). Ziebland et al [38] recently wrote of how to broaden the “Goldilocks Zone”, in which care-seeking leads to most optimal outcomes, making several suggestions: a need for the health care system to be responsive to consultations based on even diffuse symptoms; the use of ‘safety netting’ strategies to monitor symptoms until a plausible explanation is found; and better acknowledgement of practitioner uncertainty [38].

In this study, with data collected prior to the covid-19 pandemic, we note some of the challenges for study participants from three different western European countries in interpreting sensations that are often experienced as being diffuse, non-disabling, and non-threatening. These processes should have theoretical relevance in a wide range of diagnostic situations. It is also interesting to consider how these processes will be affected by the pandemic, as the sensations and symptoms in focus here, are now widely recognized as potentially indicative of covid-19. While at the time of our data collection, sensations were often diminished and not legitimized unless disabling, representatives for public health policy are now increasingly warning people of the potentially threatening nature of even the most subtle sensations. Whereas we noted that the perception of vulnerability described by Hay [22] in the sensation to symptom transformation model, appeared only indirectly in relation to threat in our data, one might thus expect this to radically change in the future.

## Conclusions

Duration, disability, and sense of threat, rather than vulnerability, were found to be relevant concepts for understanding the trajectory to diagnosis for LC among these participants, recruited from three different countries. The process by which an individual, their family and health care providers legitimize sensations, allowing them to be seen as potential symptoms of disease, is also an essential, but varying part of the diagnostic processes described here. It is of great interest to see how potential covid-19-related changes in sense of threat or vulnerability as well as legitimization processes may impact on our relationships to the types of bodily experiences described here, to healthcare seeking in the future, and to diagnosis of other disease processes with related sensations, such as LC.

## Supporting information

S1 File (DOCX)
