# Supplementary material for: Sensations, symptoms, and then what? Early bodily experiences prior to diagnosis of lung cancer
Source: PLoS One. 2021 Mar 29;16(3):e0249114. doi: 10.1371/journal.pone.0249114 (PMC8007036; doi:10.1371/journal.pone.0249114)
Supplement: S1 File — (DOCX) [file pone.0249114.s001.docx]

## Interview guide (English and Swedish)

Please begin by telling me, in your own words and in as much detail as you want, about everything that has happened since you first started to suspect there might be a problem with your health?

Kan du berätta för mig hur det har varit/hänt från det att du började misstänka att något var fel med din hälsa?

What made you suspect there was some problem with your health?

Vad fick dig att misstänka att något var fel med din hälsa?

What did you think caused the problems you experienced?

Vad trodde du var orsaken till de besvär som du kände?

Before you contacted the health care system, did you seek information about what you were experiencing? And if so, where?

Innan du sökte dig till sjukvården vart fick du tag i mer information om det du kände?

What role did your family or friends play, if any?

Var din familj eller vänner med/involverade på något sätt (hur)?

What was it that made you contact the health care system?

Vad var det slutligen som fick dig att söka vård?

What advice would you give someone else who experiences the same thing you did?

Vilka råd skulle du ge någon annan (eller vården) som har samma problem som du?

Is there anything else you would like to add that we haven’t spoken about?

Är det något mer du vill tillägga som du tror att vi skulle ha nytta av att veta som jag inte har frågat om?
